# Effects of multistep adsorption isotherms on solute transport

**DOI:** 10.1038/s41598-026-45699-4

**Published:** 2026-03-25

**Authors:** Eszter Fekete

**Affiliations:** 1 Institute of Water Resources and Environmental Management, Faculty of Earth Science and Engineering, University of Miskolc, Miskolc-Egyetemváros, 3515 Hungary; 2 National Laboratory for Water Science and Water Safety, University of Miskolc, Miskolc-Egyetemváros, 3515 Hungary

**Keywords:** Adsorption, Multistep isotherm, Stepwise, Solute transport, Porous media, Retardation, Chemistry, Engineering, Environmental sciences, Mathematics and computing, Physics

## Abstract

Multistep adsorption isotherms are characterized by sequential activation of different adsorption sites at specific threshold concentrations. This study investigates the transport behavior of solutes governed by multistep adsorption isotherms using numerical and analytical approaches. The spreading velocity of solutes is determined by the seepage velocity and the retardation factor, which itself is related to the derivative of the adsorption isotherm function. In the case of multistep isotherm functions, this derivative is not a monotonic function of solute concentration, which results in several concentration ranges with higher and lower retardation. Numerical simulations revealed how a stepwise concentration distribution develops as a result of locally decreased retardations. Preliminary relationships between isotherm parameters and transport behavior were identified by systematic parameter analysis. The analytical approach provides a more detailed understanding of the relationship between isotherm parameters and the propagation velocities as well as the criteria of stepwise concentration distribution to develop: the adsorption isotherm is composed of locally concave segments, while it displays an overall convex nature.

## Introduction

The fate and transport of solutes in porous media represents an important area of study within hydrogeology and environmental engineering. In many cases adsorption is a key factor controlling the mobility of dissolved materials, distributing it to dissolved and adsorbed phases. Reversible adsorption is generally described by isotherm equations, which provide the equilibrium relationship between the concentrations of the dissolved and the adsorbed phases. Among the most widely used parametric equations are the empirical Freundlich and the physically founded Langmuir isotherms^1,2^, which are also implemented in many solute transport simulation software^3–6^.

On a macroscopic scale, adsorption causes the retardation of dissolved components, manifesting as a delay in solute transport compared to the seepage velocity of the solvent, the water. The magnitude of retardation is determined by the slope (derivative) of the adsorption isotherm function. If this retardation is constant (which is the case for the linear isotherm function), the spatial distribution of solute concentration is not affected, only delayed. Otherwise, since different concentrations may have different spreading velocities, the concentration distribution is shaped by adsorption.

The nature of the breakthrough (or the spatial distribution of solute concentration) is determined by the curvature of the adsorption isotherm function^7^. Concave isotherm functions (e.g., the Langmuir isotherm) drive favorable adsorption. In this case the concentration dependent retardation causes a shock wave to develop at the front of the concentration distribution. This is also called compressive behavior, self-sharpening or constant pattern distribution^7–9^. If desorption occurs at the rear end of the solute plume, it is characterized by dispersive behavior (also called proportionate pattern or rarefaction zone), where a continuously decreasing concentration pattern develops due to the increasing retardation at lower concentrations. The opposite happens when dealing with convex adsorption isotherm functions. In this case, the dispersive or proportionate pattern develops at the frontal zone of a solute plume, whereas compressive or constant pattern develops at the rear end of the plume.

Some substances however exhibit more complicated behavior, which may be described by more complex isotherms, such as the multilayer BET isotherm^10,11^ or multistep adsorption isotherms^12,13^. Solute adsorption exhibiting complex isotherms has been demonstrated for many organic and inorganic compounds, such as polymers^14^, pesticides and herbicides on soils^12,13,15^ and some metals on biomass and activated charcoal substrates^16–18^. Similar behavior is well known for adsorption from gas phase^10,19^.

The effects of isotherm functions, which include both convex and concave parts, on material transport have been studied for gases using type IV and type V isotherms, commonly referred to as S-shaped isotherms^10,20,21^. These studies often describe their effect as “unusual” breakthrough^9^ and rely on numerical approaches, solving the advective-dispersive transport equation numerically, considering kinetic or equilibrium adsorption described by an S-shaped isotherm^22,23^.

However, the way these multistep adsorption isotherms are affecting solute transport and are manifesting in a stepwise concentration distribution and corresponding breakthrough curves has not been thoroughly studied. In the present work the effect of multistep isotherms (as defined by Konda et al.^12^ and Czinkota et al.^13^ on solute transport is being described. In some cases these isotherms can express a dual nature according to their curvature: locally concave with and overall convex nature. One important feature of these multistep isotherms is that their first derivative is not a monotonic function, hence the retardation factor may have several peaks if plotted against the solute concentration. This feature may manifest in a stepwise concentration distribution with several smaller shock waves following each other.

The current study focuses on the effects of multistep isotherms. First, numerical simulations are being carried out in order to gain an initial intuition of the transport process. Then, an analytical approach is being adapted to multistep isotherms to further clarify our understanding of how these isotherms influence solute transport.

The aims of the study are as follows:

- identify how the parameters of multistep isotherm functions affect solute transport.
- give an accurate description of the relationship between the isotherm properties and the solute transport behavior.
- define the condition of stepwise concentration distribution to develop.

## Materials and methods

Both numerical and analytical methods have been applied to simulate solute transport controlled by retardation caused by multistep adsorption isotherms. The numerical approach relies on a modified version of MT3DMS. In summary, eight simulation test series were run, each for the inspection of the effect of one single isotherm parameter. While the numerical approach provides a first intuition of how the multistep adsorption isotherm affects solute transport, the analytical approach provides the exact relationship. The results of the analytical approach have been compared with the results obtained by numerical simulation.

### Multistep adsorption isotherms

To describe the complex adsorption behavior observed for certain contaminants, this study utilized the multistep isotherm function Eq. (1), defined by Czinkota et al.^13^. This model introduced limit concentrations above which a new adsorption mechanism starts to take place. The total adsorption is conceptualized as the sum of several individual adsorption processes, each becoming active above a specific threshold or limit concentration. The model is formulated as a summation of modified Langmuir-like expressions:

1 $$\:q=f\left(c\right)=\sum\:_{i=1}^{N}\frac{{S}_{i}{K}_{i}\left[c-{b}_{i}+abs\left(c-{b}_{i}\right)\right]}{2+{K}_{i}\left[c-{b}_{i}+abs\left(c-{b}_{i}\right)\right]}$$

where $\:q$ [M/M] and $\:c$ [M/L3] are the concentrations in the adsorbed and solute phases, respectively. $\:{b}_{i}$ [M/L3] is the limit concentration of the i^th^ isotherm step. $\:{S}_{i}$ [M/M] and $\:{K}_{i}$ [L3/M] are the Langmuir parameters of the i^th^ isotherm step, where $\:{S}_{i}$ is the saturation concentration of the adsorbed phase, and $\:{K}_{i}$ is a coefficient related to the equilibrium distribution. N is the number of isotherm steps, and $\:f$ is solely being used to denote the adsorption isotherm function.

For simplicity, Eq. (1) may be rewritten into a form composed of several simple Langmuir isotherms.

2 $$\:q=f\left(c\right)=\sum\:_{i=1}^{N}\frac{{S}_{i}{K}_{i}{C}_{i}^{*}}{1+{K}_{i}{C}_{i}^{*}}$$

3 $$\:{C}_{i}^{*}=\frac{1}{2}\left[c-{b}_{i}+abs\left(c-{b}_{i}\right)\right]$$

**Fig. 1:**
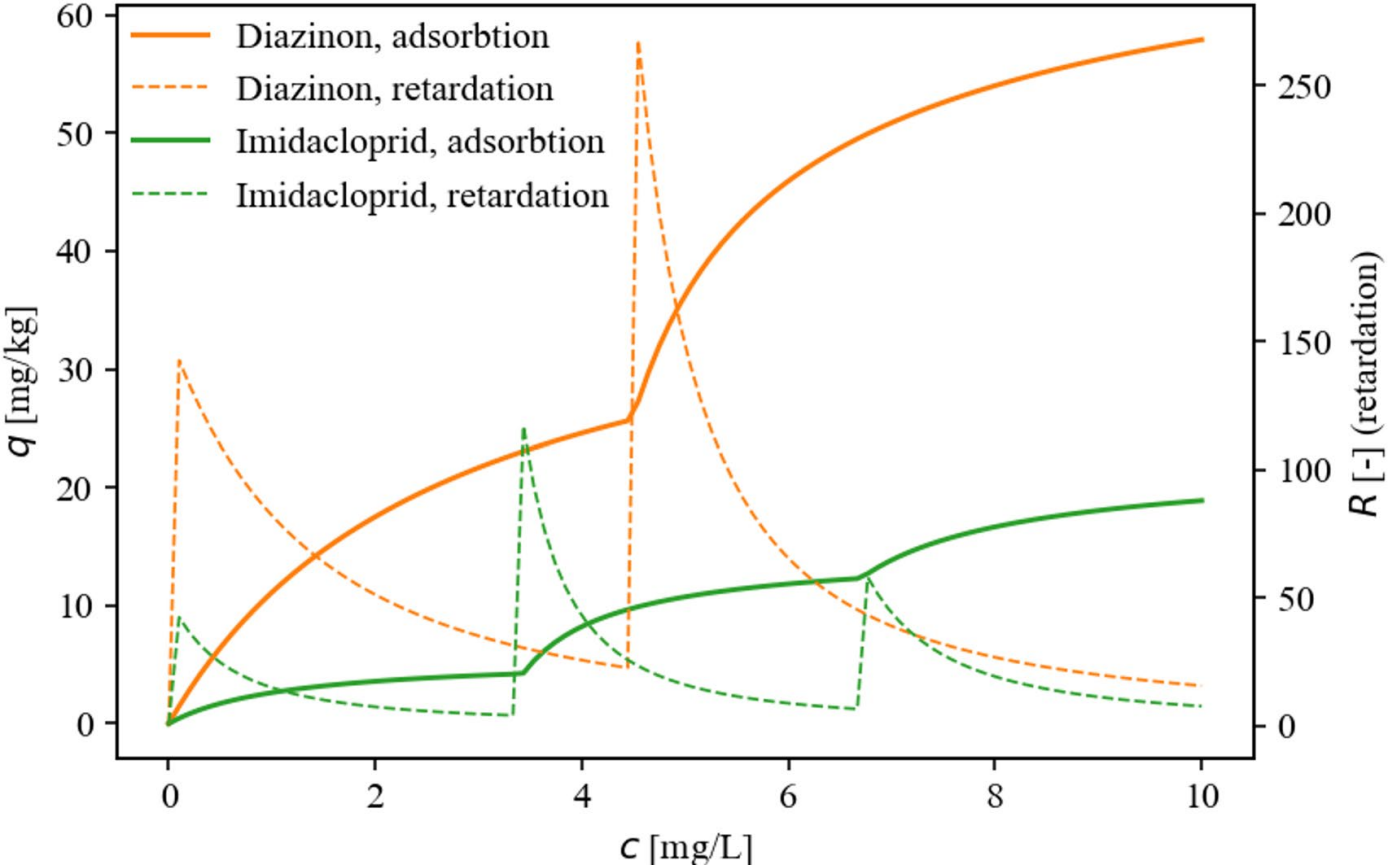
Examples for adsorption isotherms and concentration dependent retardations published in Konda et al. 2002.

In this notation $\:{C}_{i}^{*}$ is the effective concentration value, given by the exceedance above the limit concentration. This can be interpreted as a local $\:c$ value for the i^th^ isotherm step. The purpose of this formula is to ensure that $\:{C}_{i}^{*}$ is greater than zero only if $\:c>{b}_{i}$, as used by Czinkota et al.^13^. For the published cases, the value of $\:{b}_{1}$ is equal to zero. Figure 1 shows two examples of multistep adsorption isotherms published by Konda et al.^12^ and the corresponding retardation functions.

### Numerical approach

To gain insight into the transport behavior of multistep adsorption isotherms, 1D numerical simulations were conducted. The simulations relied on a modified version of the MT3DMS Version 5.30^24,25^, which extends the original RCT package. MT3DMS solves the advective-dispersive transport equation for dissolved phases also taking into account adsorption and possible first-order chemical reactions and sources:

4 $$\:\frac{\partial\:nc}{\partial\:t}+\rho\:\frac{\partial\:q}{\partial\:t}=\frac{\partial\:}{\partial\:x}\left(nD\frac{\partial\:c}{\partial\:x}\right)-\frac{\partial\:n{v}_{w}c}{\partial\:x}+W$$

where $\:n$ [-] and $\:\rho\:$ [M/L3] are the porosity and bulk density of the media respectively; $\:D$ [L2/T] is the dispersion/diffusion coefficient; $\:{v}_{w}$ [L/T] is the average linear flow velocity of water flow (the Darcy velocity divided by the porosity); and $\:W$ represents source/sink terms as well as chemical reaction terms.

A 1D uniform flow field was used for the advective term. Dispersion and diffusion are not considered in the simulation. Their effect would act toward equilibrating the concentration differences along the flow. No other chemical reactions were considered.

By applying the above simplifications (neglecting dispersion/diffusion and ignoring sources and chemical reactions) on a uniform and steady flow field, the transport equation reduces to:

5 $$\:n\frac{\partial\:c}{\partial\:t}+\rho\:\frac{\partial\:q}{\partial\:t}=-n\frac{\partial\:{v}_{w}c}{\partial\:x}$$

The lefthand side may be further simplified by assuming local equilibrium between the adsorbed and dissolved phases and expressing the material balance only in terms of dissolved concentration:

6 $$\:\frac{\partial\:c}{\partial\:t}\left(1+\frac{\rho\:}{n}\frac{\partial\:q}{\partial\:c}\right)=-\frac{\partial\:{v}_{w}c}{\partial\:x}$$

where $\:\partial\:q/\partial\:c$ is the derivative of the adsorption isotherm. The term in the parentheses is called retardation (usually denoted with $\:R$ [-]) and gives the ratio of the average linear velocity ($\:{v}_{w}$) of water flow and the solute transport velocity ($\:{v}_{s}$):

7 $$\:\frac{{v}_{w}}{{v}_{s}}=R=1+\frac{\rho\:}{n}\frac{\partial\:q}{\partial\:c}$$

### Modifications of MT3DMS

The RCT package of MT3DMS is limited to the use of linear, Langmuir and Freundlich adsorption isotherms. As part of this study, the MT3DMS source code was modified in order to incorporate the use of the multistep adsorption isotherm defined by Eq. (1). As an alternative approach, the possibility to define adsorption isotherms by $\:c$-$\:q$ data pairs was also included. In this case piecewise, linear or spline interpolation is used to calculate the $\:\partial\:q/\partial\:c$ ratio and the retardation. In this manner any arbitrary adsorption isotherm curve can be defined and used. This option was used to validate the numerical simulation of multistep isotherms defined by isotherm parameters.

**Fig. 2:**
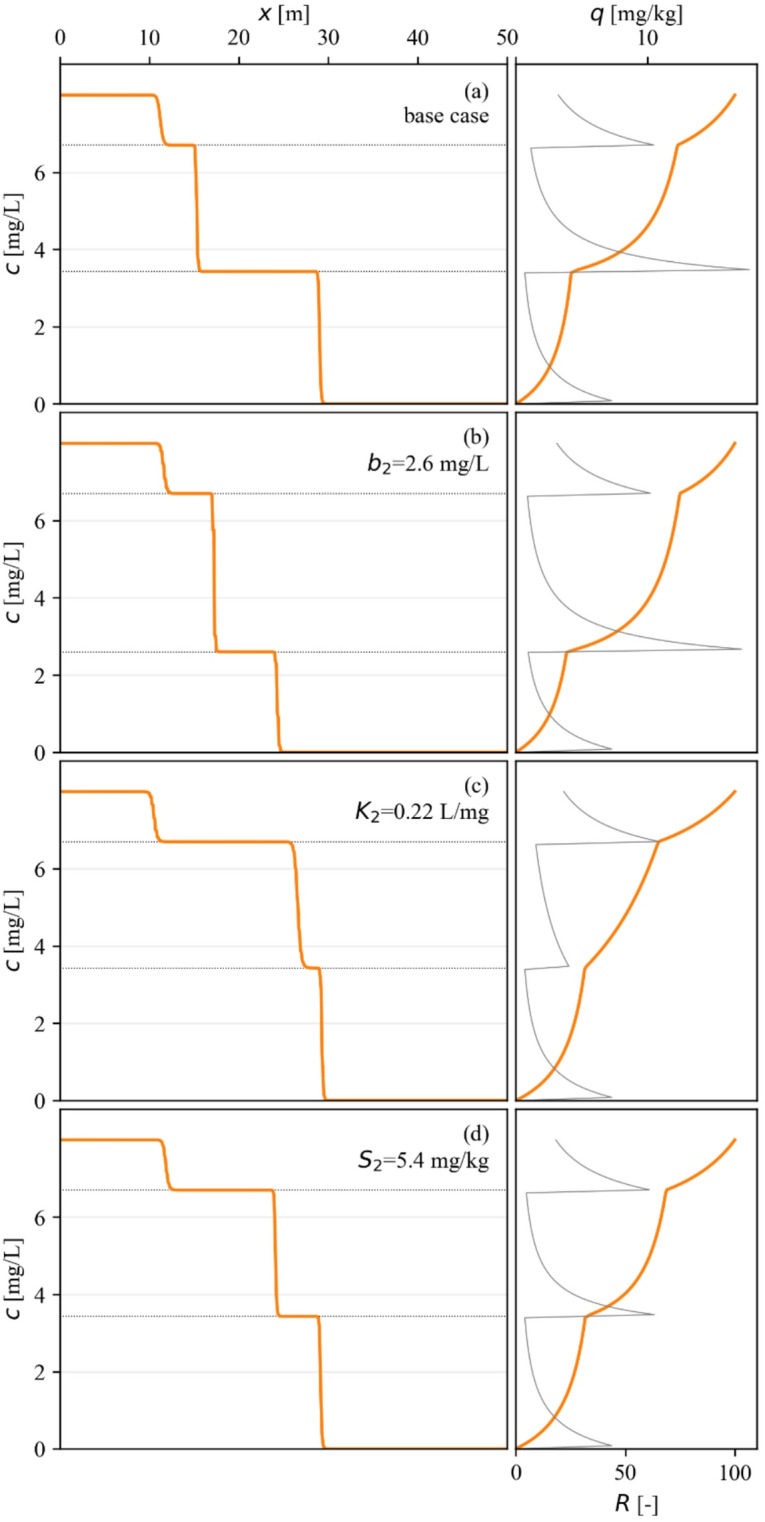
Effect of changing of isotherm parameters on dissolved and sorbed phase concentrations and retardation values.

**Fig. 3:**
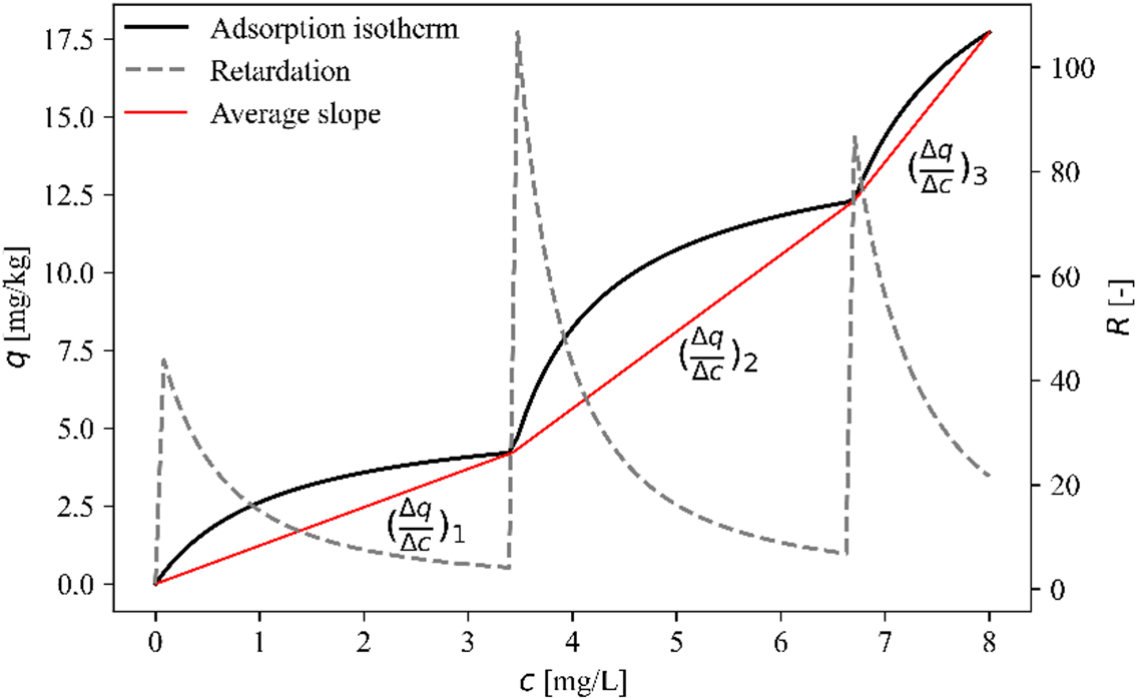
Plot of a multistep isotherm indicating the overal slopes (red lines) and retardation values (dashed lines).

The concentration-dependent retardation factor ($\:R$) may be obtained by the derivative of the isotherm equation, Eq. (1) with respect to the concentration. The following equation was used in the modified MT3DMS version to calculate retardations as well as to plot them on the figures of the paper (Figs. 1 and 2, and 3):

8 $$\:\frac{{v}_{w}}{{v}_{s}}=R=1+\frac{\rho\:}{n}\frac{\partial\:q}{\partial\:c}=1+\frac{\rho\:}{n}\sum\:_{i=1}^{N}\frac{{K}_{i}{S}_{i}}{{(1+{K}_{i}{C}_{i}^{*})}^{2}}$$

### Simulation runs

By using the modified numerical code, a systematic parameter study was performed to explore the relationship between the isotherm parameters and the solute transport behavior.

For the numerical simulations, a one-dimensional grid composed of 500 cells, 0.1 m each, with a total extent of 50 m was used. A steady-state uniform flow field (generated by MODFLOW 2005 v1.12.00^26^ was used with a Darcy velocity of 0.01 m/day (corresponding to a hydraulic conductivity of 1 m/day with a hydraulic gradient of 0.01). The porous material was assumed to have a bulk density of 2 kg/L and a porosity of 0.2. Adsorption was simulated with the modified RCT package of MT3DMS, assuming equilibrium adsorption determined by the presented multistep isotherm. The dispersion and diffusion was neglected in order to focus on the effect of the given multistep adsorption isotherms.

Initial and boundary conditions were set up to simulate the shock front propagation with a continuous source. Zero initial concentration was specified for the whole domain, and a constant concentration source (set to 8 mg/L) was applied at the inflow boundary. The constant concentration boundary was essential since the propagation velocity of the shock fronts depends on the solute concentration, and particularly on the peak concentration. Without the constant concentration source, the peak concentrations would decrease over time. The effect of the peak concentration was later clarified by the analytical approach.

Advection was simulated by using the TVD scheme available in MT3DMS, supported by its superior performance in situations where sharp concentration fronts are present^24^, which is the case for solute transport involving nonlinear adsorption. Despite the better performance of MOC scheme under purely advective transport without adsorption^27^, preliminary benchmark simulations executed with simple Langmuir adsorption isotherm reflected that the TVD scheme is more reliable for this kind of problems. Preprocessing and postprocessing were performed with the FloPy framework^28^, which is a powerful tool for running batch simulations and data processing needed for the systematic parameter study.

During the systematic parametric study, the parameters of a three-step adsorption isotherm were changed one at a time, keeping the other properties constant. The adsorption isotherm of imidacloprid published by Konda et al.^12^ was selected as the base case. The parameters of the base case are listed in Table 1, and the isotherm defined by them is plotted in Fig. 3. For each of the eight parameters ($\:{K}_{1}$, $\:{K}_{2}$, $\:{K}_{3}$, $\:{S}_{1}$, $\:{S}_{2}$, $\:{S}_{3}$, $\:{b}_{2}$ and $\:{b}_{3}$) eleven equally spaced values have been tested. The ranges of the parameters were defined to include the base case values in the central part of the range. The parameter values used for the systematic parametric study are listed in Table 2. The $\:K$ parameter varied from 0.1 to 2 L/mg, while the base case values were 0.87, 1.22 and 0.73 L/mg. The $\:S$ parameter varied from 3 to 11 mg/kg, while the base case values were 5.64, 9.36 and 7.79 mg/kg. When the range of the $\:b$ parameters was selected, it was considered not to overlap the adjacent $\:b$ values. For example, the range of $\:{b}_{3}$ (3.81–7.62 mg/L) does not extend to the value of $\:{b}_{2}$ (3.43 mg/L), neither to the value of the constant concentration source (8 mg/L).

**Table 1:** Isotherm parameters of the initial case, as published by Konda et al.^12^.

	$\:{\boldsymbol{K}}_{\boldsymbol{i}}$ [L/mg]	$\:{\boldsymbol{S}}_{\boldsymbol{i}}$ [mg/kg]	$\:{\boldsymbol{b}}_{\boldsymbol{i}}$ [mg/L]
$\:\boldsymbol{i}=1$	0.87	5.64	0
$\:\boldsymbol{i}=2$	1.22	9.36	3.43
$\:\boldsymbol{i}=3$	0.73	7.79	6.70

**Table 2:** Values of isotherm parameters used the simulation test series.

Test case	$\:{\boldsymbol{K}}_{1}$, $\:{\boldsymbol{K}}_{2}$, $\:{\boldsymbol{K}}_{3}$ [L/mg]	$\:{\boldsymbol{S}}_{1}$, $\:{\boldsymbol{S}}_{2}$, $\:{\boldsymbol{S}}_{3}$ [mg/kg]	$\:{\boldsymbol{b}}_{2}$ [mg/L]	$\:{\boldsymbol{b}}_{3}$ [mg/L]
0	0.1	3	0.56	3.81
1	0.29	3.8	1.12	4.19
2	0.48	4.6	1.68	4.57
3	0.67	5.4	2.23	4.95
4	0.86	6.2	2.79	5.33
5	1.05	7	3.35	5.72
6	1.24	7.8	3.91	6.1
7	1.43	8.6	4.47	6.48
8	1.62	9.4	5.03	6.86
9	1.81	10.2	5.58	7.24
10	2	11	6.14	7.62

For each simulation, the propagation velocities of the concentration fronts have been determined by tracking the movement of the front center over time. According to the initial and boundary conditions, no distinct concentration fronts were present at the beginning of the simulations, and a transitional period was needed for the fronts and steps to develop. This period was characterized by fluctuations in the obtained propagation velocities, a characteristic consistently observed at the beginning of every simulation. These early time fluctuations were excluded from the velocity calculations.

### Analytical approach

To complement the numerical simulations and provide a deeper theoretical understanding, an analytical approach was employed on the basis of previous developments. The first analytic solutions of the advective transport equation involving nonlinear adsorption were developed by Thomas^29^ and Hiester and Vermeulen^30^. A description of the concentration shock front behavior was given by Tondeur et al.^7^ and van der Zee^31^, and later extended by Sheng and Smith^32^ with the rarefaction behavior. The solutions described by these latter two were adapted for the cases of multistep adsorption isotherms.

According to their solution, the propagation velocity of the concentration front ($\:{v}_{F}$ [L/T]), if it develops due to nonlinear adsorption, is given by:

9 $$\:{v}_{F}=\frac{1}{{R}_{F}}{v}_{w}$$

10 $$\:{R}_{F}=1+\frac{\rho\:}{n}\frac{\varDelta\:q}{\varDelta\:c}$$

where $\:\varDelta\:q$ and $\:\varDelta\:c$ are the concentrations of the sorbed phase and dissolved phase on the two sides of the concentration front, respectively, and their ratios yield an average slope of the adsorption isotherm function $\:q=f\left(c\right)$. Therefore the propagation velocity is determined by the inverse of this average slope. A high average slope contributes to slow front propagation.

If this is applied to a multistep adsorption isotherm, separate propagation velocities are to be determined for each isotherm step. The numerical simulations revealed that the “height” of each concentration step is determined by the $\:{b}_{i}$ limit concentrations, where the next isotherm step starts to manifest. Therefore, the $\:\varDelta\:c$ of the first concentration front is simply given by $\:{b}_{2}-{b}_{1}$, whereas $\:\varDelta\:q$ can be read from the isotherm, being equal to $\:f\left({b}_{2}\right)-f\left({b}_{1}\right)$. It can be noted that for the used isotherms both $\:{b}_{1}$ and $\:f\left({b}_{1}\right)$ are actually equal to zero. For the i^th^ concentration front $\:\varDelta\:c={b}_{i+1}-{b}_{i}$ and $\:\varDelta\:q=f\left({b}_{i+1}\right)-f\left({b}_{i}\right)$.

In case of n concentration fronts the *n*+1th limit concentration is not defined by the adsorption isotherm; instead, the maximum concentration is to be used. In the current situation it is equal to the value set for the inlet concentration.

## Results and discussion

Results are presented in a consequent order: starting with the results of the numerical simulations, the introduction of step-wise distribution of a solute plume, and the parameter study. Then the analytical approach is presented and compared to the numerical results to validate it.

### Numerical approach

#### Formation and propagation of stepwise concentration fronts

**Fig. 4:**
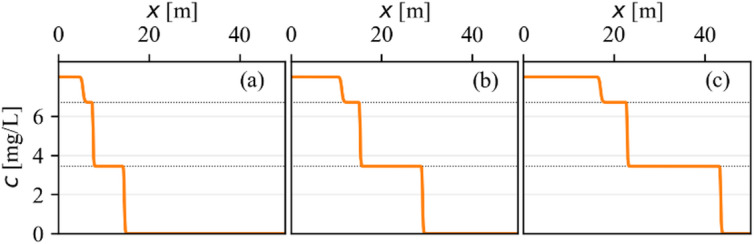
Stepwise concentration distribution along flow direction as a result of multistep isotherm. The three plots represents three times t_1_=4000 days, t_2_=2t_1_, t_3_=3t_1_. The dotted horizontal lines mark the limit concentrations.

A distinguished trait of solute transport governed by a multistep adsorption isotherm is the development of a distinct, stepwise concentration distribution. As depicted in Fig. 4, a single, sharp concentration front introduced at the inlet rapidly evolves into a series of cascading steps. An analogy may be drawn with the coherence principle of multicomponent wave propagation^33,34^. An initial noncoherent wave is split into smaller coherent waves, which remain stable unless additional disturbances occur. Each plateau in the concentration profile corresponds directly to the limit concentrations ($\:{b}_{i}$) of the underlying multistep isotherm, in this case at 3.43 mg/L and 6.70 mg/L, with the final step reaching the inflow concentration of 8 mg/L.

Figure 4 illustrates the temporal evolution of this distribution at three sequential time points ($\:{t}_{1}=4000$ days, $\:{t}_{2}=2{t}_{1}$, and $\:{t}_{3}=3{t}_{1}$). The subplots clearly show that once formed, the concentration steps maintain their respective concentration levels and propagate through the domain at constant, but different, velocities. The lowest concentration front (at 3.43 mg/L) travels the fastest, followed by the intermediate front (at 6.70 mg/L), with the highest concentration front (at 8.0 mg/L) traveling the slowest. This velocity relation is the mechanism driving the separation of the plume into distinctly propagating steps. For example, between times $\:{t}_{1}$ and $\:{3t}_{1}$, the first front travels approximately 28 m, whereas the third front travels only approximately 11 m, demonstrating a significant difference in propagation speed.

#### Influence of isotherm parameters on transport behavior

The influence of individual isotherm parameters on transport behavior was investigated through systematic parameter analysis, with the first results shown in Fig. 2. This figure compares the base case scenario (Fig. 2a) with simulations where the parameters of the second isotherm step ($\:{b}_{2}$, $\:{K}_{2}$, and $\:{S}_{2}$) were individually modified (Fig. 2b, c and d). All four plots were created for simulation time $\:t={t}_{2}$ from Fig. 4. The right-hand panels of the figures illustrate the corresponding adsorption isotherms and retardation factor curves determined by the original and modified parameters.

The limit concentration parameter, $\:{b}_{i}$ directly controls the concentration value of the corresponding step plateau. As shown in Fig. 2b, modifying the value of $\:{b}_{2}$ from 3.43 mg/L to 2.6 mg/L results in a corresponding shift of the intermediate concentration plateau to the new value. This finding directly confirms that the step heights are governed by the threshold concentrations at which new adsorption mechanisms are activated. The propagation velocities of the first and second concentration fronts are also significantly affected. The first one is slightly decreased, while the second one is increased.

In contrast, the $\:{K}_{i}$ and $\:{S}_{i}$ parameters influence only the propagation velocity of the fronts, and not their concentration levels. In Fig. 2c, a decrease in $\:{K}_{2}$ (from 1.22 L/mg to 0.22 L/mg) primarily affects (increases) the velocity of the second front with a minor impact (decrease) on the third front and no impact on the first front. This is also visible on the curve of the retardation factor, where the second peak is considerably reduced. A similar effect is observed in Fig. 2d for a modification of $\:{S}_{2}$ (decreasing from 9.36 mg/kg to 5.4 mg/kg), where the propagation velocity of the second front increases significantly and that of the third front increases to a lesser extent. This localized influence demonstrates that the transport behavior of a specific concentration step is most strongly controlled by the $\:{K}_{i}$ and $\:{S}_{i}$ parameters that define that step. The $\:{K}_{i}$ and $\:{S}_{i}$ parameters belonging to the step below have only negligible influences. On the other hand, both the threshold concentrations ($\:{b}_{k}$) that belong to the given k^th^ step and the one above it ($\:{b}_{k+1}$) may have a strong influence on propagation velocities.

#### Systematic analysis of front velocities

**Fig. 5:**
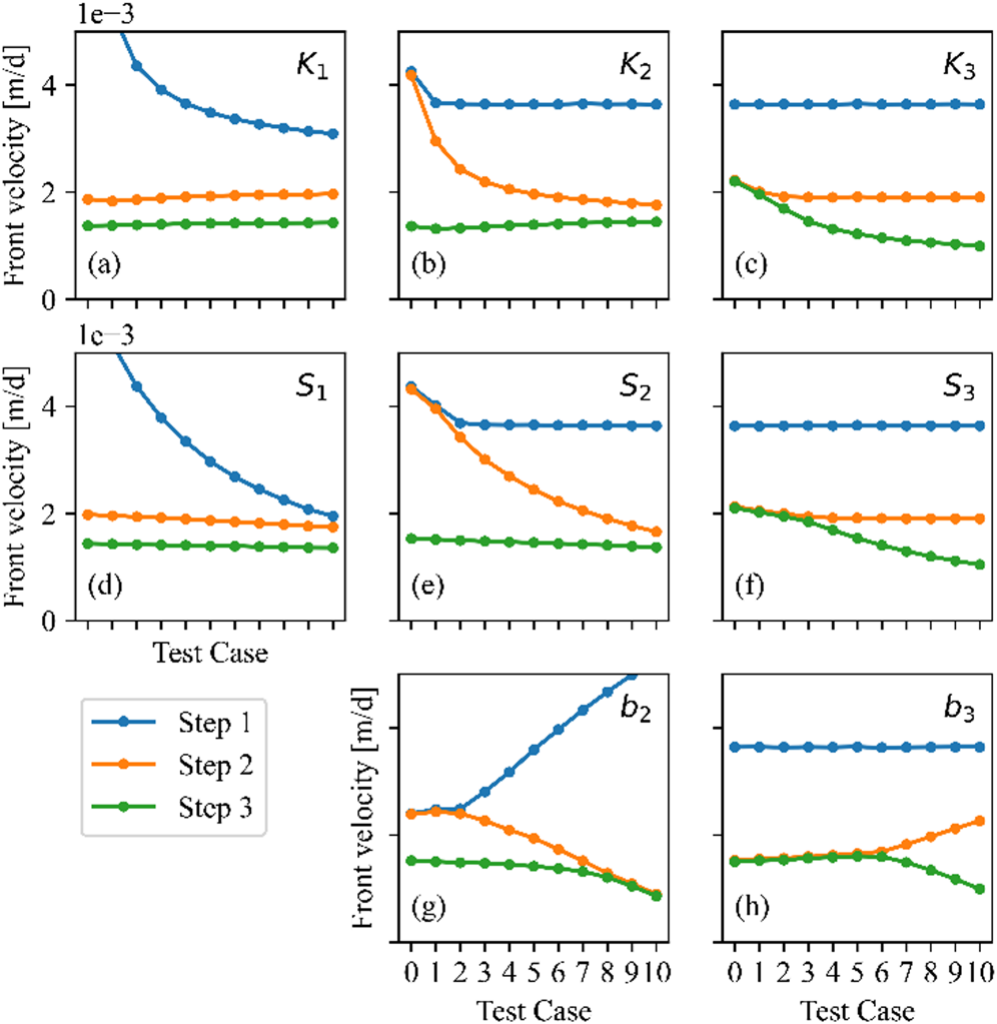
Effect of different isotherm parameters (K_1_, K_2_, K_3_, S_1_, S_2_, S_3_, b_2_, b_3_) on the spreading velocity of the three concentration fronts.

Figure 5 provides a comprehensive overview of the parameter analysis, plotting the propagation velocities of the three concentration fronts as a function of the 11 test cases for each of the eight key isotherm parameters. Some important observations can be made based on it. First, an increase in the parameter value consistently leads to a decrease in the propagation velocity of the corresponding front. This is expected, as higher affinity or capacity leads to greater retardation. Second, the influence of the $\:{K}_{i}$ and $\:{S}_{i}$ parameters is largely localized to their own step; for example, varying $\:{K}_{2}$ or $\:{S}_{2}$ has a negligible effect on the velocity of the third front (green line) and no effect on the first front (blue line). The limit concentration parameters ($\:{b}_{i}$) have a more complex influence, affecting the velocities of both their own and the preceding fronts. For example, increasing $\:{b}_{2}$ not only decreases the velocity of the second front (orange line) but also increases the velocity of the first front (blue line). This is because a change in $\:{b}_{2}$ alters the concentration range ($\:\varDelta\:c$) over which the first front’s average slope is calculated.

In most cases three distinct concentration fronts and the associated steps develop. However, in some cases, the merging of two steps occurs when the propagation velocities of two adjacent fronts become equal. For example, in the test cases for $\:{K}_{2}$, low values cause the velocities of the first and second steps to converge, resulting in a single, merged front. The parameter values clearly determine which steps tend to merge, as seen for the $\:{b}_{2}$ parameter, where for small values the first two steps are merged. By increasing the parameter value, these merged steps first separate, and then the middle step starts to get closer to the upper step, until these two steps finally merge into a single step.

### Analytical solution and theoretical framework

The analytical approach provides clear theoretical insights into the conditions required for stepwise concentration distribution development and validates the numerical findings.

#### Criteria for stepwise front formation

Figure 3 illustrates the fundamental theoretical principle governing stepwise front formation. The figure demonstrates that the overall concavity of the multistep isotherm breakpoints (shown as red lines) exhibits a globally convex character, despite each individual step being locally concave. This dual nature is the key to understanding when stepwise fronts develop and when they remain separated.

Theoretical considerations reveal that stepwise concentration distributions can only develop when the propagation velocity of lower concentration steps exceeds that of higher concentration steps ($\:{v}_{1}>{v}_{2}>{v}_{3}$). On the basis of the analytical approach described in the section “Analytical approach”, this velocity relation is ensured when the average slopes of the isotherm segments, calculated as $\:{\left(\varDelta\:q/\varDelta\:c\right)}_{i}$, increase with each successive step. As shown in Fig. 3, the slopes of the red connecting lines increase from $\:{\left(\varDelta\:q/\varDelta\:c\right)}_{1}$ to $\:{\left(\varDelta\:q/\varDelta\:c\right)}_{3}$, creating the necessary condition for front separation. When this condition is not met, adjacent fronts merge into a single propagating step, as observed in some parameter combinations in Fig. 5.

This finding provides a predictive criterion for determining whether a given multistep isotherm will produce distinct, separated fronts or merged concentration steps. The criterion can be expressed mathematically as:

$$\:{\left(\varDelta\:q/\varDelta\:c\right)}_{1}<{\left(\varDelta\:q/\varDelta\:c\right)}_{2}<\dots\:<{\left(\varDelta\:q/\varDelta\:c\right)}_{n}$$

#### Validation of the analytical approach

**Fig. 6:**
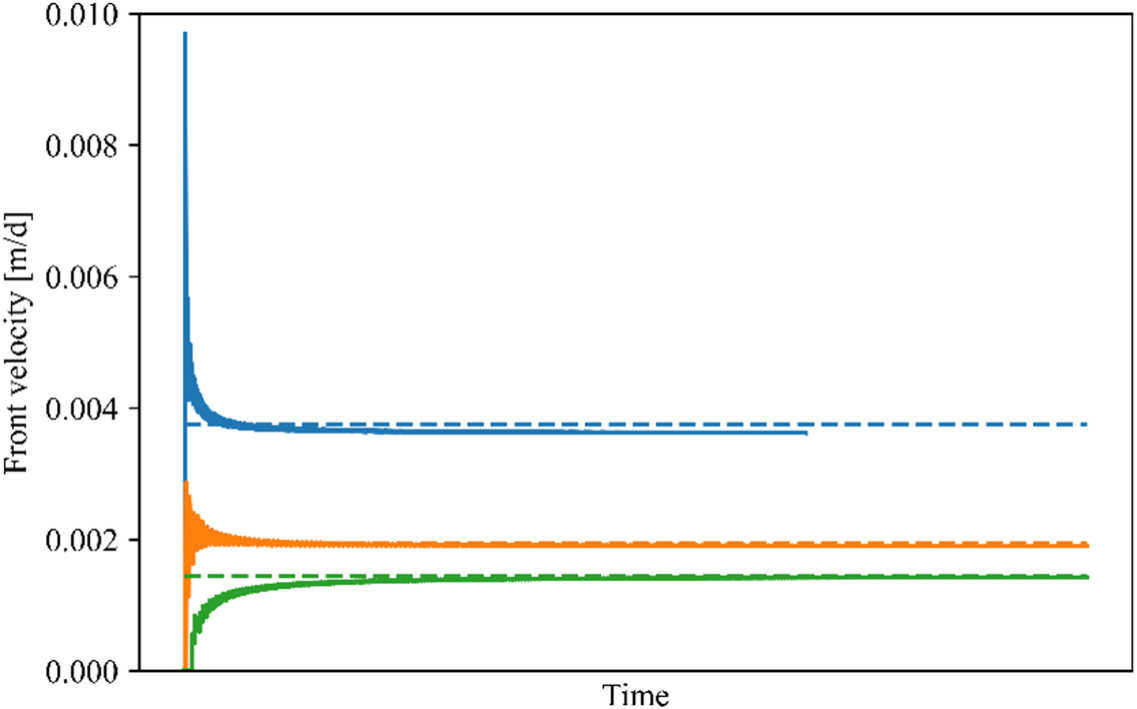
Comparison of front velocities obtained by numerical simulation (continuous line) and the analytical approach (dashed line) for the base case isotherm.

Figure 6 shows the comparison of the front velocities for the base case obtained from the numerical simulation results (continuous lines) with the analytical predictions (dashed lines). Since a constant source boundary is applied and the initial concentration is zero, the numerical model takes some time for the distinct concentration steps to form in an isotherm-controlled transport regime. This is observed on the initial part, where velocity fluctuations occur. The fluctuations are neglected during the average calculation. Once steady velocities are achieved, the analytical predictions match the numerical results with good accuracy. It is noted that the fastest front eventually reaches the downstream boundary and exits the domain, while the slower fronts continue propagating until the end of the simulation.

**Fig. 7:**
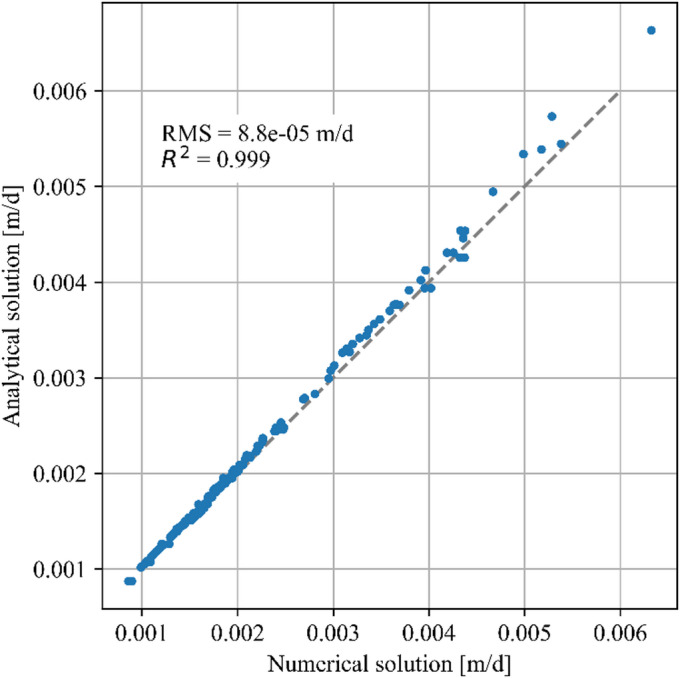
Comparison of average front velocities obtained by numerical simulation and the analytical approach for each test cases.

Figure 7 shows the comparison of the analytically and numerically obtained velocities across the complete parameter space. The data points emerge from the 3 concentration fronts tested in 88 simulation runs (for 8 isotherm parameters with 11 test values each). Among the 264 obtained front velocities, front merges occurred in 21 cases. The results demonstrate good agreement between the two methods, with a correlation coefficient (R^2^) of 0.99 and a root mean square (RMS) error of 8 × 10^− 5^ m/d. The discrepancies are assumed to be caused only by numerical inaccuracies. The excellent agreement of the obtained values proves the reliability of the two methods used.

## Discussion

The results of this study provide a clear and reliable explanation for the formation of stepwise concentration distribution during solute transport when it is controlled by multistep adsorption isotherms. This behavior is a consequence of the nonmonotonic nature of the retardation factor as a function of concentration. Contrary to the Langmuir or Freundlich isotherms, for which retardation is a monotonic function of concentration, multistep isotherms may lead to a series of retardation peaks, corresponding to each adsorption step (Fig. 3). Concentrations higher than the limit are characterized by sharply increased retardation, which delays their propagation. This also explains the coincidence of the number of steps as long as some criteria on the isotherm are met. The distinct concentration ranges with decreased transport velocities, cause an initially coherent plume to separate into multiple fronts.

Furthermore, the curvature of the isotherm has also to be considered. The development of self-sharpening shock fronts is characteristic of concave isotherms (e.g., Langmuir), where retardation decreases with increasing concentration. In the studied multistep isotherm, each individual step can be viewed as a locally concave region, contributing to the formation of a stable shock front. However, the overall nature of the adsorption isotherm is convex globally, as the average slope increases with each successive step, which leads to a dispersive front pattern. This dual nature of the isotherm curvature is responsible for the observed stepwise transport by locally creating stable shock fronts while globally sustaining the needed velocity differences. This scale-dependent duality is a novel theoretical concept in solute transport.

The effects of isotherm parameters are mostly localized to the corresponding concentration front, however, some cross relationships also exist. The $\:{b}_{i}$ parameter has a direct influence on the concentration front one below, by affecting both $\:\varDelta\:c$ and $\:\varDelta\:q$ of that step. The slight influences of the $\:{b}_{i}$, $\:{K}_{i}$ and $\:{S}_{i}$ parameters on the upper concentration fronts are caused by the summary nature of the multistep isotherm function used. Since one given isotherm step still has a small but existing slope in the range of the following isotherm step, it gives an additional slope alongside the steps’ own slope. This type of cross relationship of isotherm steps may be solely an artifact of the multistep isotherm equation, which is defined as the sum of single isotherm steps.

The adapted analytical approach used in this study provides a quantitative framework of solute transport controlled by multistep adsorption isotherms. Previous studies of complex isotherms, such as the work by Inglezakis and Fyrillas^23^ on S-shaped isotherms, relied primarily on numerical approaches and provided only qualitative descriptions of transport behavior. The current work advances beyond these descriptive approaches by providing explicit mathematical relationships between isotherm parameters and front velocities, predictive criteria for determining when fronts will separate or merge and quantitative tools for calculating arrival times of multiple concentration levels.

Application of the presented framework can provide new perspectives in the field of environmental remediation and separation processes. The use of the adequate adsorption isotherm in solute transport modeling can improve the reliability of the results and the efficiency of remediation techniques designed upon them. It was demonstrated that concentration fronts can have very different propagation velocities as a result of the multistep nature of the isotherm. Even in the base case, the differences expressed as ratios are 2.5 in magnitude. For several other test cases the ratios can be even higher (see Fig. 4). The implementation of the multistep adsorption isotherm in MT3DMS makes it possible to apply it for 3D real-world problems. The drawback of using multistep adsorption isotherm is the increased data demand, compared to single Langmuir or linear isotherms. Parametrization of the multistep isotherms are measurement-intensive and is further complicated by the possible heterogeneity of real-world systems.

Although the current study presents a comprehensive approach to the topic, future studies should address some of the remaining issues. In the present study the formulated theoretical framework was validated against numerical simulations, which itself were validated against more simple transport models using single Langmuir isotherms. Laboratory measurements of solute breakthrough in column tests would provide a solid experimental validation of the presented theoretical framework. Generalization of the presented framework could be achieved by investigating the trailing rarefaction behavior for both isotherms similar to the one used in this study and isotherms with an overall concave nature built of convex steps (as opposed to the ones used in the current study). The concentration distribution developed by multistep isotherms may be altered by other transport processes such as dispersion/diffusion or reactions. Another research direction could be the study of the effects of dispersion and/or diffusion on the sharp concentration fronts, especially with respect to the exponent of the concentration values in the Czinkota isotherm, as both terms tend to smooth out the sharp changes in concentrations.

## Conclusions

Previous studies revealed that some solutes can exhibit more complex adsorption isotherms than that a single-step isotherm model can describe. Czinkota et al^13^. suggested a multistep isotherm model for those instances, composed of a sum of single-step isotherms sequentially activated at given threshold concentrations. In this study the influence of multistep isotherms composed of Langmuir isotherms on solute transport behavior was investigated. The soundness of the outcomes is demonstrated by the good agreement between the results obtained with the numerical and the analytical approaches.

It was found that under certain conditions these isotherms cause a stepwise concentration distribution to develop. The systematic parameter analysis conducted by numerical simulations and the applied analytical approach revealed the following: (1) the threshold concentrations of the isotherm directly determine the values of the concentration steps; (2) the propagation velocities of the concentration fronts depend on the combined effects of the isotherm parameters. The exact relationship between the propagation velocities and the isotherm parameters was explained by the analytical approach of adapting existing solutions to multistep isotherms. The propagation velocity of each concentration front is determined by the average slope of the isotherm function over the given step. Clearly, the criterion of distinct concentration fronts to develop is that their propagation velocities are lower and lower for higher concentrations. It can be concluded that these criteria demands a multistep isotherm with increasing overall slopes, which leads to an overall convex nature but is still composed of concave Langmuir segments.

## Data Availability

All datasets are detailed in the paper and the cited sources. Source code can be obtained from the author.
